# Distinct Metabolic Profile of Primary Focal Segmental Glomerulosclerosis Revealed by NMR-Based Metabolomics

**DOI:** 10.1371/journal.pone.0078531

**Published:** 2013-11-11

**Authors:** Xu Hao, Xia Liu, Weiming Wang, Hong Ren, Jingyuan Xie, Pingyan Shen, Donghai Lin, Nan Chen

**Affiliations:** 1 Nephrology Department, Ruijin Hospital, Shanghai Jiao Tong University School of Medicine, Shanghai, China; 2 Bimolecular NMR Laboratory, Shanghai Institute of Materia Medica, Chinese Academy of Sciences, Shanghai, China; 3 The Key Laboratory for Chemical Biology of Fujian Province, College of Chemistry and Chemical Engineering, Xiamen University, Xiamen, China; Fondazione IRCCS Ospedale Maggiore Policlinico & Fondazione D’Amico per la Ricerca sulle Malattie Renali, Italy

## Abstract

**Background:**

Primary focal segmental glomerulosclerosis (FSGS) is pathological entity which is characterized by idiopathic steroid-resistant nephrotic syndrome (SRNS) and progression to end-stage renal disease (ESRD) in the majority of affected individuals. Currently, there is no practical noninvasive technique to predict different pathological types of glomerulopathies. In this study, the role of urinary metabolomics in the diagnosis and pathogenesis of FSGS was investigated.

**Methods:**

NMR-based metabolomics was applied for the urinary metabolic profile in the patients with FSGS (n = 25), membranous nephropathy (MN, n = 24), minimal change disease (MCD, n = 14) and IgA nephropathy (IgAN, n = 26), and healthy controls (CON, n = 35). The acquired data were analyzed using principal component analysis (PCA) followed by orthogonal projections to latent structure discriminant analysis (OPLS-DA). Model validity was verified using permutation tests.

**Results:**

FSGS patients were clearly distinguished from healthy controls and other three types of glomerulopathies with good sensitivity and specificity based on their global urinary metabolic profiles. In FSGS patients, urinary levels of glucose, dimethylamine and trimethylamine increased compared with healthy controls, while pyruvate, valine, hippurate, isoleucine, phenylacetylglycine, citrate, tyrosine, 3-methylhistidine and β-hydroxyisovalerate decreased. Additionally, FSGS patients had lower urine N-methylnicotinamide levels compared with other glomerulopathies.

**Conclusions:**

NMR-based metabonomic approach is amenable for the noninvasive diagnosis and differential diagnosis of FSGS as well as other glomerulopathies, and it could indicate the possible mechanisms of primary FSGS.

## Introduction

Focal segmental glomerulosclerosis (FSGS) was first described in kidney biopsy of adults with nephrotic syndrome by Fahr in 1925, and in 1957 Rich observed that sclerosis classically start from the corticomedullary junction before involving other parts of the renal cortex in children with nephrotic syndrome (NS) [1]–[3]. FSGS is a pathological entity which is characterized by idiopathic steroid-resistant nephrotic syndrome (SRNS) and progression to end-stage renal disease (ESRD) in the majority of affected individuals. The current gold standard for the pathological diagnosis of FSGS is renal biopsy, which is invasive and has poor repeatability in monitoring progression of disease. Therefore, a noninvasive approach for FSGS diagnosis is desirable. Regarding the pathogenesis, several mechanisms have been found associated with predisposing and progression of FSGS which include podocyte depletion or changes [4]–[6], hemodynamics, hyperlipidemia, glomerular visceral epithelial cells (GVEC) damages, cellular immunity and glomerular permeability factor (GPF), *etc*, while the exact mechanisms of FSGS remains unknown. The recent emergence of systems biology provides new insight into diagnosis and pathogenesis of diseases.

Metabolomics is defined as “the quantitative measurement of the dynamic multiparametric metabolic response of living systems to pathophysiological stimuli or genetic modification” [7]. Metabolomics could be used in the diagnosis of critically ill patients [8], human bladder cancer [9], prostate cancer [10] and so on. Any perturbation to a living system will cause fluctuation of concentration or flux of endogenous metabolites. Therefore, we suspected that there might be unique metabolic profile in patients with FSGS. Under this assumption, we analyzed metabolites in urine from patients with primary FSGS, speculated metabolic profile of disease and further explored the underling mechanisms.

In the present study, we performed NMR-based metabolomics to distinguish the distinct metabolic profile of primary FSGS from other primary glomerulopathies [MN, MCD, and IgAN] and healthy controls. Subsequently, we evaluated the potential diagnostic value of this new approach in differential diagnosis of primary glomerulopathies and gained novel insights into the pathogenesis. Our study indicated several metabolic pathways which involved in the pathogenesis of primary FSGS.

## Materials and Methods

### Ethics Statement

This study was approved by the Institutional Review Board of the Ruijin Hospital, Shanghai Jiao Tong University School of Medicine and was carried out according to the Principles in the Helsinki Declaration II. The written informed consent was obtained from each participant.

### Selection and Description of Participants

All the participants with primary glomerulopathies (FSGS, MN, IgAN and MCD) and healthy volunteers were recruited in Ruijin Hospital, Shanghai Jiao Tong University School of Medicine. Two experienced pathologists reviewed all the kidney biopsies. The inclusion criteria are as follows: 1) Biopsy-proven primary glomerulopathy; 2) Patients with new-diagnosed primary glomerulopathy and haven’t received any drug therapy (ACE-I/ARB, steroids, immunosuppressants, etc.); 3) Patients at CKD stages 1 to 4. The diagnosis and CKD stage were determined based on the criteria of the National Kidney Foundation (NKF). The glomerular filtration rate (GFR) was estimated by the equation from the study “Modification of Diet in Renal Disease” (MDRD) [11]. The exclusion criteria are as follows: 1) Obesity; 2) Reflux nephropathy; 3) HIV-associated nephropathy; 4) Malignant cancers; 5) Autoimmune diseases; 6) Infection; 7) Hereditary kidney diseases; 8) History of alcoholism and continued smoking.

### Sample Collection and Preparation

All the participants were fasting and water deprivation after 8∶00 pm on the day before renal biopsy. Urine and blood samples were obtained before breakfast on the day of the renal biopsy. Urine samples were collected at the second excretion in solid CO_2_-cooled tubes (15****mL) containing 250 µL of 1% (w/v) sodium azide. Aliquots of urine (500 µL) obtained from each participant were centrifuged at 4000×g for 10 min at 4°C and then stored at −80°C before NMR analysis. Blood was allowed to clot at room temperature and centrifuged to separate the upper serum. Urine samples were used in metabonomic analysis, and serum samples were used in biochemistry examination.

An aliquot of urine (300 µL) was mixed with 300 µL of phosphate buffer (0.2 M Na_2_HPO_4_/0.2 M NaH_2_PO_4_, pH 7.4) in order to minimize pH variations. The mixture was vortexed for 2 min and then centrifuged at 12,000 rpm for 10 min at 4°C. The supernatants (500 µL) were transferred into 5 mm NMR tubes and then added 50 µL of D_2_O containing 0.01% sodium 3-(trimethylsilyl) [2, 2, 3, 3-D4] propionate (TSP).

### ^1^H NMR Spectroscopy of Urine

^1^H NMR spectra were acquired at 25°C on a Varian Unity INOVA 600 MHz spectrometer equipped with three RF channels and a triple resonance z-axis pulsed-field gradient probe. For a large of protein was present in urine samples of CKD patients, transverse relaxation-edited spectra were recorded to attenuate the broad NMR signals of slowly tumbling molecules with short T_2_ relaxation times and to retain signals of low-molecular weight compounds. The water-suppressed Carr-Purcell-Meiboom-Gill (CPMG) pulse sequence [RD-90°-(τ-180°-τ)_n_-ACQ] was used here. A fixed total spin-spin relaxation delay 2 nτ of 120 ms was used. Water suppression irradiation was applied during the relaxation delay (5 s). Typically, 128 FIDs were collected into 32,768 data points using a spectral width of 10 kHz with an acquisition time of 1.64 s.

### Statistical Methods

All 1D FIDs were multiplied by an exponential function of a 0.3 Hz line-broadening factor prior to Fourier transformation. The NMR spectra were manually phased, and baseline corrected. The chemical shifts were referenced to the methyl group of TSP at δ 0.00 for the spectra of urine. All the 1D NMR spectra were carefully aligned by MestReNova software (Version 6.2, Mestrelab Research S.L.). The spectral region of δ 9.50-0.50 was segmented into 3000 bins with a width of 0.003 ppm. The integrals from the region of δ 6.00-4.67were excluded to eliminate distorted baseline from imperfect water saturation in all spectra. Urinary creatinine is considered as a “house-keeping metabolite”, integrals of bins in every sample were subtracted by the level of bin including the urinary creatinine. The integrals were normalized using the approach of probabilistic quotient normalization to compensate for differences in sample concentrations [12]. Subsequently, the normalized integral values were variable autoscaled for orthogonal partial least-squares discriminant analyses (OPLS-DA) by the software package SIMCA-P+ (Version 12.0, Umetrics, Umeå, Sweden). As a supervised approach, OPLS-DA between two groups was calculated with the first predictive (t [1]) and one orthogonal component (to [1]), however, the OPLS-DA model with the five groups (CON, FSGS, MN, IgAN and MCD) were autofitted. The terms R^2^ and Q^2^ were used to evaluate the quality of OPLS-DA models. R^2^ are the fraction of variance in the data explained by the model and indicates goodness of fit. Q^2^ represents the cross-validated explained variation and indicated predictability. The standard 7-round cross validation and permutation test (999 cycles) on the first predictive component was carried out to measure the robustness of the model.

Variable importance in the projection (VIP) derived from the OPLS-DA model ranks the importance of each variables for the classification, and those variables with VIP>1.0 are initially considered statistically significant in this model. The correlation coefficients (r) of the variables relative to the first model score value in the OPLS-DA model were also extracted from S-plot calculated by Pearson correlation. Cutoff values of r with a significant level of 0.05 were used to identify variables that were responsible for the discrimination of groups. Thus the integrals of metabolites which were meeting VIP>1 and the correlation coefficients |r|>the critical value of p = 0.05 may account for the discrimination.

To evaluate the predictive ability of the OPLS-DA models for new samples, the predictive sets as no class samples will show in the scatter plot of OPLS-DA training sets. The first predictive component is used as the boundary, and the distribution of no class samples are observed in the OPLS-DA score plots. The sensitivity of the models is calculated from the ratio between true positive and the total number of modeled FSGS spectra, whereas specificity is determined from the ratio between the true negative and the total number of modeled control and other glomerulopathies spectra.

### Univariate Statistics of Metabolites’ Integral

Group means of metabolites’ integral are expressed as the mean±std. The median and interquartile range of metabolites in each group is also showed. The analysis of the comparison of the mean values among the FSGS, IgAN, MN, MCD, the subgroups of FSGS and CON groups was performed utilizing one way analysis of variance (ANOVA) (Bonferroni test for post hoc multiple comparison). The statistical analysis was performed using SPSS software (Version 17.0, SPSS, Inc.). *p*<0.1 was considered statistically significant. The *q* value and FDR were established with the ANOVA *p* values or the student’s ttest *p* value by the script has been described in the supplementary material.

## Results

### 1 Clinical Parameters

89 primary glomerulopathy patients (FSGS, n = 25; MN, n = 24; IgAN, n = 26; MCD, n = 14) and 35 healthy controls were enrolled. 61.4% patients were at CKD1, 15.7% at CKD2, 18.1% at CKD3 and 4.8% at CKD4. The distribution of CKD stages in different groups has no statistical significance. All the participants enrolled in the present study were first-visit patients and no patients had hypertension. Clinical parameters and the full lipid profile were shown in Table 1 and Table 2 respectively. Patients with MCD had the highest level of 24-hour urinary albumin excretion compared with the patients with other glomerulopathies. In addition, patients with MN and MCD had a higher level of serum cholesterol and lower albumin than that in FSGS and IgAN. Statistically, there were no significant differences for other clinical parameters among different types of glomerulopathies (Table 1, Table 2).

**Table 1:** Biochemical parameters of the subjects used in this study.

Pathology	Age(years)	Gender(F/M)	BMI	SBP(mm Hg)	DBP(mm Hg)	GFR(ml/min)	BUN(mmol/l)	Alb(g/L)	Glc(mmol/l)	24 hrUprV(mg)
Health	40.75±16.23	18/17	23.5±2.8	116.8±10.9	75.2±6.9	119.8±17.6	4.87±1.2^&^	41.88±3.11	4.29±0.42^†^	<30
FSGS	40.09±13.15	12/13	23.9±3.4	120.5±11.9	77.5±9.4	94.1±33.1*	6.2±2.15*^†^	36.05±7.76*^†‡^	4.73±0.88*^#‡^	1095(50–5769) *^†‡^
IgAN	37.8±11.74	13/13	22.4±2.7	124.2±13.1	79.3±9.4	98.9±22.3*	5.76±2.58	34.9±4.67*^†‡^	4.36±0.47^‡^	1171.5(77–4574)*^†‡^
MN	45.6±11.52	14/10	23.4±1.9	116.7±10.7	75.8±7.8	108.6±45.3	4.77±1.63^&^	20±7.21*	4.8±0.71*	3155(181–9502)*
MCD	30.11±14.46	8/6	21.9±1.8	125.7±12.5	78.3±8	101.1±32.4*	4.81±5.56	22.63±11.2*	3.9±0.6^†^	4849(3196–5629)*^†^

BMI: body mass index; SBP: systolic blood pressure; DBP: diastolic blood pressure; GFR: glomerular filtration rate; BUN: blood urea nitrogen; Alb: albumin; Glc: glucose; 24 hrUprV: protein amount of 24 hours urine.

P*<0.05 versus healthy controls,

P^&^<0.05 versus FSGS,

P^#^<0.05 versus IgAN,

P^†^<0.05 versus MN,

P^‡^<0.05 versus MCD.

**Table 2:** Full lipid profile of different group (CON, FSGS, IgAN, MN and MCD).

Pathology	TG(mmol/L)	TC(mmol/L)	HDL	LDL
Health	0.82(0.56–1.7)	4.41(3.11–5.91)	1.32(0.92–1.75)	2.88(1.35–4.28)
FSGS	2.21(0.51–5.52)*	5.2(3.83–10.12)*^†‡^	1.26(0.82–1.85)	3.34(2.01–7.12)*^ ‡^
IgAN	1.24(0.45–8.17)*	4.92(3.34–8.92)*^†‡^	1.34(0.81–2.38)	3.18(1.41–5.5)*^ ‡^
MN	2.46(0.8–2.46)*	7.33(3.29–14.5)*	1.36(0.99–2.55)	4.86(1.81–10.19)*^ ‡^
MCD	2.66(1.02–7.36)*	9.62(3.93–13.89)*	1.75(1.13–2.49)	6.82(2.4–10.71)*

TG: triglyerides; TC: total cholesterol; HDL: high density lipoprotein; LDL: low density lipoprotein.

P*<0.05 versus healthy controls,

P^†^<0.05 versus MN, P^‡^<0.05 versus MCD.

### 2 Results of NMR-based Metabolomics

#### 2.1 Metabolic alterations in urine samples by 1H-NMR of FSGS and CON

**Figure 1:**
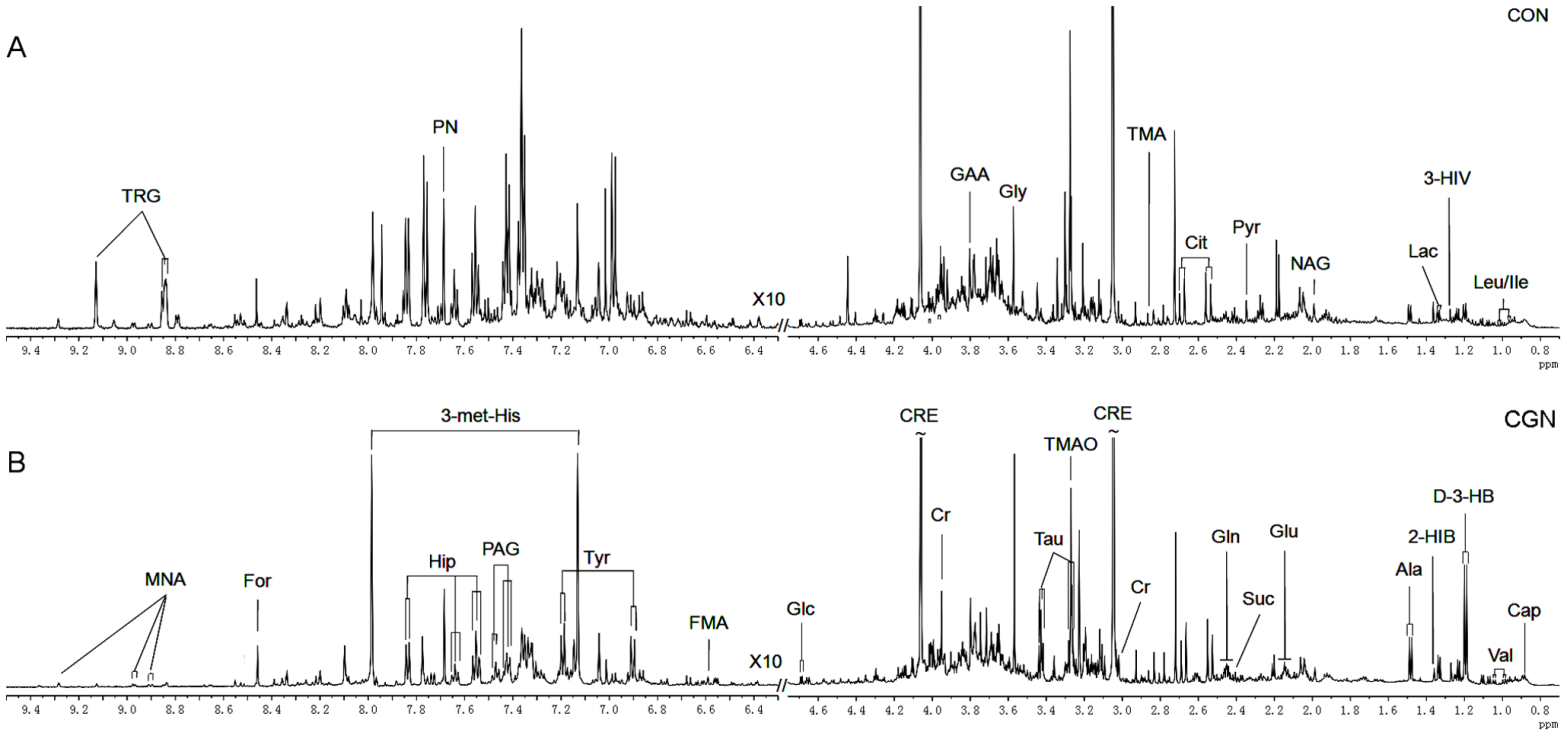
Representative 600 MHz 1H NMR spectra of urine from control people and patients with FSGS. All these marked metabolites were the metabolite variables in the present work.

Figure 1 shows representative spectra from the urine of FSGS patients and healthy controls. Visual inspection of the spectra reveals the different excretive profiles in various urine metabolites between FSGS and CON. Metabolites that were commonly observed in the urine spectrum from the healthy individuals and the patients with FSGS are creatinine (CRE), hippurate (Hip), citrate (Cit), trimethylamine-N-oxide (TMAO), dimethylamine (DMA), trimethylamine (TMA), pyruvate (Pyr), glucose (Glc), taurine (Tau), 3-hydroxybutyrate (3-HB), 3-methylhistidine(3-me-His), N-acetyl groups from glycoproteins (NAC), 3-hydroxyisovalerate (3-HIV), trigonelline (TRG), N-methylnicotinamide (NMN), 2-hydroxyisobutyrate (2-HIB), phenylacetylglycine(PAG), and amino acids such as alanine (Ala), isoleucine (Ile), tyrosine (Tyr), valine (Val), glycine (Gly), glutamine (Glu), glutamate (Gln), and formate (For). The integral value of most significant metabolites in healthy control, FSGS, IgAN, MCD and MN patients obtained from 1H NMR spectra was shown in Table S1. The median and interquartile range of the metabolites in each group was shown in Table S2.

#### 2.2 Pattern recognition analysis between FSGS, MCD, MN, IgAN and CON

**Figure 2:**
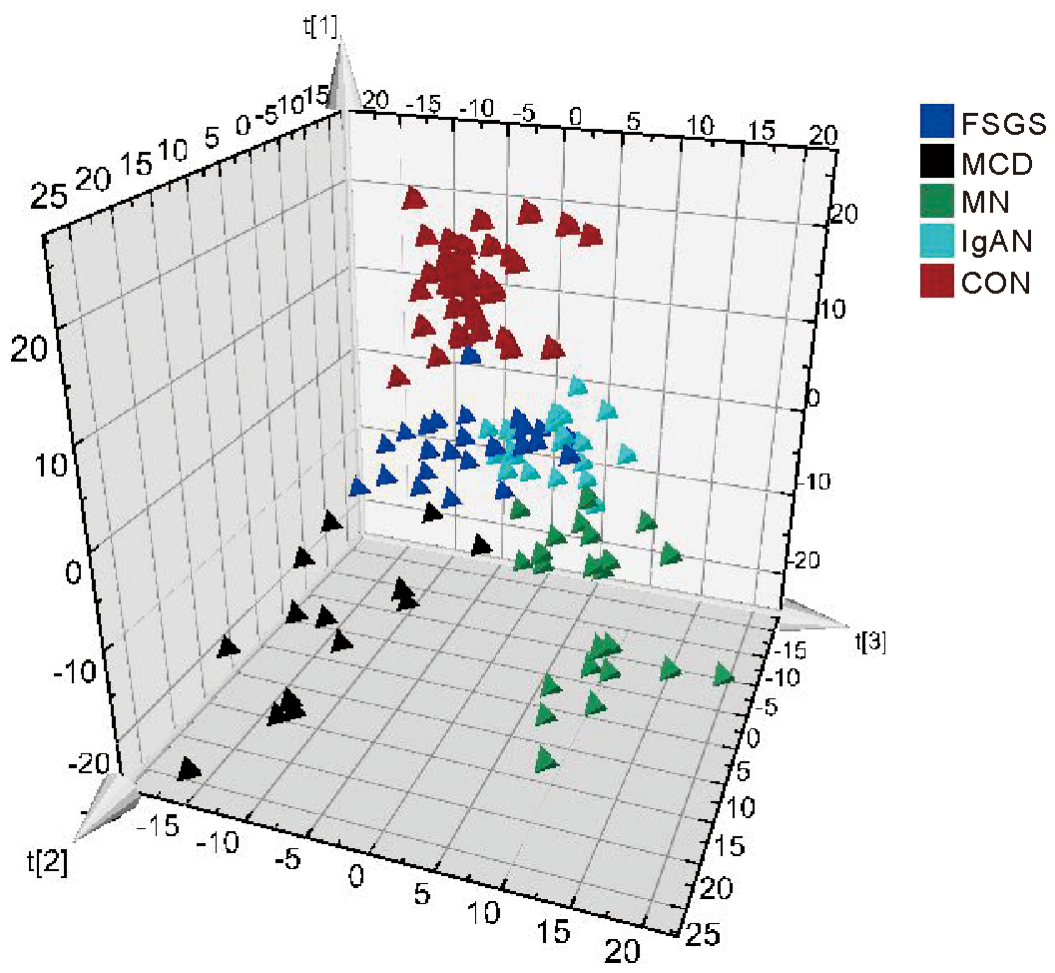
OPLS-DA scores plot of urine 1H NMR spectra of healthy controls and patients with FSGS, IgAN, MN and MCD.

For the NMR data of 89 patients and 35 healthy individuals, OPLS-DA was applied, and the scores plot (Figure 2) revealed diverse trends among patients in different etiological groups. Healthy individuals were clustering to the top section, MCD and MN segregated mainly to the bottom-left and bottom-right sections, respectively. In spite of slight overlapping, a separation was also achieved between FSGS and IgAN. The PLS-DA model for different group was shown in Figure S1.

#### 2.3 The separation of FSGS and CON

**Figure 3:**
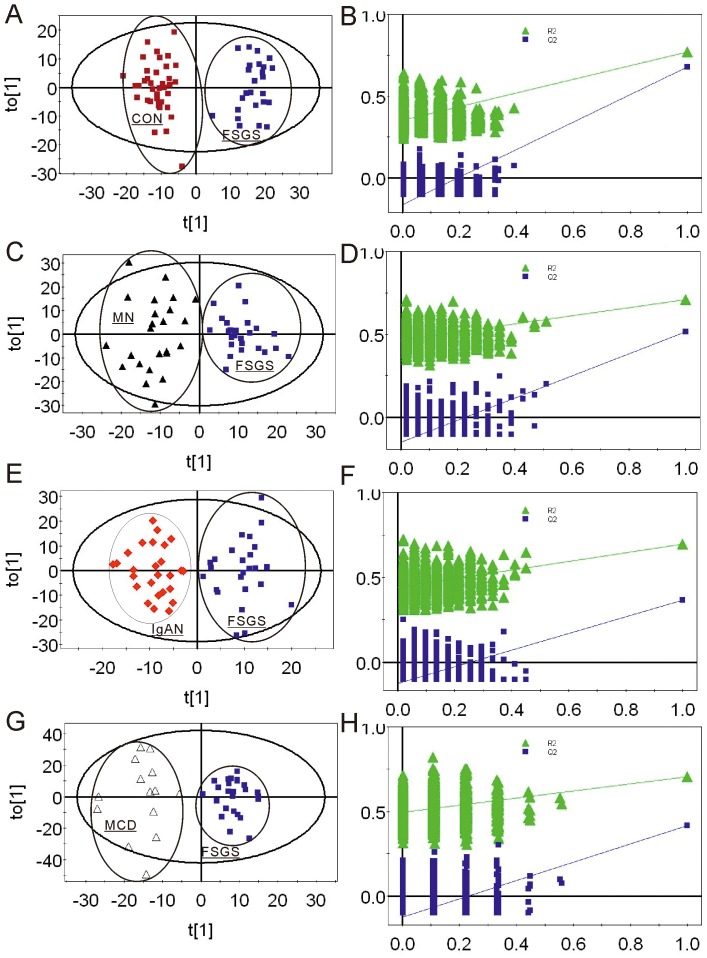
OPLS-DA scores plot and validation of the OPLS-DA model using a permutation test of urine 1H NMR spectra of FSGS and CON (A, B), FSGS and MN (C, D), FSGS and IgAN (E, F), FSGS and MCD (G, H).

An orthogonal projection to latent-structure (OPLS) analysis was run to discriminate the FSGS patients from healthy people. The urine spectra were well discriminated with the OPLS model with a Q^2^ of 0.776 and a R^2^ of 0.920 (Table 3), as shown in Figure 3A with the good cross-validation of permutation tests (Figure 3B). The VIPs and p(corr) extracted from the OPLS-DA plot and the values of p and q obtained from one way ANOVA also revealed patients with FSGS mainly characterized by high levels of Glc, DMA, TMA, and decreased excretion of Pyr, Hip, PAG, Ile, Val, Tyr, Cit, 3-HIV and 3-me-His (Table 4,5).

**Table 3:** The parameter values of OPLS-DA models.

OPLS-DA models	R^2^	Q^2^
FSGS-CON	0.92	0.776
FSGS-MCD	0.876	0.529
FSGS-IgAN	0.777	0.516
FSGS-MN	0.83	0.538

**Table 4:** The integral change trends in FSGS vs. CON, FSGS vs.MN, FSGS vs. IgAN, FSGS vs. MCD, and the values of q, p and FDR.

metabolites	integral change trends			
	Average change ofFSGS vs. CON(p/q)	Average change ofFSGS vs.MN(p/q)	Average change ofFSGS vs. IgAN (p/q)	Average change ofFSGS vs. MCD (p/q)
Glc	+77.9%(0.002,0.003)	−30.6%(0.087,0.087)	/(1.000,–)	−34.6%(0.032,0.038)
Pyr	−59.4%(0.000,0.000)	+24.4%(0.077,0.077)	/(1.000,–)	+36.8%(0.005,0.015)
Val	−21.1%(0.000,0.000)	−16.2%(0.007,0.01)	/(0.498,–)	/(0.918,–)
Hip	−76.0%(0.000,0.000)	+107.6%(0.028,0.028)	/(0.978,–)	/(0.989,–)
DMA	+18.6%(0.000, 0.000)	/(0.270,–)	/(0.966,–)	+36.8%(0.020,0.030)
TMA	+98.9%(0.010,0.011)	/(1.000,–)	/(1.000,–)	/(1.000,–)
Ile	−22.3%(0.003,0.004)	/(0.746,–)	/(1.000,–)	/(1.000,–)
PAG	−30.3%(0.025,0.025)	/(1.000,–)	/(1.000,–)	/(1.000,–)
Cit	−35.6%(0.000,0.000)	−25.9%(0.041,0.041)	/(0.660,–)	/(0.776,–)
TMAO	/(0.942,–)	/(1.000,–)	+39.1%(0.024,0.048)	/(0.299,–)
NAC	/(0.689,–)	/(1.000,–)	/(0.849,–)	/(0.967,–)
PN	/(0.746,–)	/(0.750,–)	/(0.944,–)	+20.6%(0.071,0.071)
Ala	/(1.000,–)	/(1.000,–)	/(0.996,–)	/(1.000,–)
Tau	/(0.194,–)	/(0.907,–)	+27.5%(0.081,0.081)	/(0.803,–)
3-HB	/(0.259,–)	/(0.661,–)	−32.5%(0.038,0.051)	/(0.238,–)
NMN	/(0.591,–)	−42.5%(0.088,0.088)	−47.2%(0.023,0.048)	−60.4%(0.001,0.006)
TRG	/(0.114,–)	/(0.394,–)	/(0.149,–)	−47.8%(0.018,0.036)
Leu	/(0.824,–)	/(0.592,–)	/(1.000,–)	/(1.000,–)
IB	/(0.226,–)	/(1.000,–)	/(1.000,–)	/(1.000,–)
3-HIV	−24.4%(0.001,0.002)	/(0.811,–)	/(0.784,–)	/(0.876,–)
Lac	/(1.000,–)	/(1.000,–)	/(1.000,–)	/(1.000,–)
2-HIB	/(1.000,–)	/(1.000,–)	/(1.000,–)	/(1.000,–)
Lys	/(0.197,–)	/(0.970,–)	/(1.000,–)	/(1.000,–)
AA	/(1.000,–)	/(1.000,–)	/(1.000,–)	/(1.000,–)
Suc	/(0.979,–)	/(1.000,–)	/(0.628,–)	/(0.595,–)
Gln	/(1.000,–)	/(0.991,–)	/(0.993,–)	/(1.000,–)
Gly	/(0.857,–)	/(1.000,–)	/(0.999,–)	/(0.293,–)
Cr	/(1.000,–)	/(1.000,–)	/(0.293,–)	/(1.000,–)
3-me-His	−45.3%(0.000,0.000)	/(0.775,–)	/(0.940,–)	/(1.000,–)
Tyr	−44.3%(0.000,0.000)	/(0.771,–)	/(1.000,–)	/(1.000,–)
FDR	0.008	0.040	NA	0.017

The changes of metabolites with q and/or p value less than 0.10 are considered as statistical significance; NA means no FDR value been calculated; “–” stands for no q value been calculated; The value of q and FDR were calculated using the function of fdr tool packages in the R environment (programming language).

**Table 5:** Metabolite assignment of integral fragments statistically important for the separation of FSGS from CON, MN, IgAN and MCD.

Metabolites	FSGS vs CON		FSGS vs MN		FSGS vs IgAN		FSGS vs MCD	
	VIP	r	VIP	r	VIP	r	VIP	r
Glc	1.73	+0.56	1.30	−0.38	/	/	2.01	−0.50
Pyr	1.66	−0.64	1.8	+0.5	/	/	1.67	+0.36
Val	1.56	−0.47	1.64	−0.43	/	/	/	/
Hip	1.56	−0.53	1.65	+0.43	/	/	/	/
DMA	1.54	+0.49	/	/	/	/	1.54	+0.49
TMA	1.44	+0.52	/	/	/	/	/	/
Ile	1.37	−0.45	/	/	/	/	/	/
PAG	1.37	−0.55	/	/	/	/	/	/
Cit	1.07	−0.31	1.37	−0.34	/	/	/	/
Ala	/	/	1.83	−0.51	/	/	/	/
NMN	/	/	1.62	−0.42	2.22	−0.45	1.76	−0.39
TRG	/	/	1.24	−0.39	1.79	−0.38	1.27	−0.34
TMAO	/	/	/	/	2.28	+0.52	/	/
Tau	/	/	/	/	1.72	+0.37	1.58	+0.40
3-HB	/	/	/	/	1.61	−0.33	/	/
NAC	/	/	/	/	1.31	−0.29	/	/
PN	/	/	/	/	/	/	1.45	+0.33

The cutoff value of r is respectively ±0.254, ±0.282, ±0.276 and ±0.316.

“/” means values of VIP<1 or | r |<| cutoff |.

Because of the differences in proteinuria and eGFR between FSGS patients and healthy controls, subgroup analysis was performed to eliminate the influence of the two factors. According to the proteinuria, FSGS patients were divided into two subgroups (the cutoff value of proteinuria was 1500****mg/24 h); according to the eGFR, FSGS patients were divided into another two subgroups (CKD1+2 and CKD3+4). From the results of the PCA and OPLS-DA scatter plots (Figure S2, Figure S3), healthy controls could be distinguished from each subgroup while the subgroups could not be distinguished from each other. In addition, for the metabolites (Table S3), most of the metabolites are the same with the Table 4. Therefore, the results of the subgroup analysis indicted that proteinuria and eGFR partly contributed to the discrimination and the underling pathogenesis of the disease was considered the most important influential factor of the metabolic profile.

#### 2.4 The separation of FSGS and MN

To identify the special metabolic characteristics of FSGS patients, the pairwise OPLS-DA models were established among FSGS, MN, IgAN, and MCD. The OPLS-DA scores plots showed a significant separation between FSGS and MN groups (Figure 3 C, D, Table 3). The model parameter for the explained variation R^2^ was 0.83 and the predictive capability Q^2^ was 0.538 which suggested the robustness of our model. On the basis of the VIPs, p(corr), p and q extracted from the OPLS-DA models, patients with FSGS mainly excreted higher levels of Hip, and Pyr, whereas patients with MN mainly excreted higher levels of Cit, NMN, Glc, and Val. (Table 4,5).

#### 2.5 The separation of FSGS and IgAN

A distinct separation was observed between FSGS and IgAN (R^2^ = 0.777 and Q^2^ = 0.516) (Figure 3 E, F, Table 3). On the basis of the values of VIPs, p(corr), p and q, the metabolites that predominantly contributed to the separation of the FSGS from the IgAN group were higher levels of TMAO and Tau, and lower levels of NMN and 3-HB, (Table 4,5).

#### 2.6 The separation of FSGS and MCD

As it is indicated by the R^2^ and Q^2^ parameters of the OPLS-DA model, 0.876 and 0.529 (Figure 3 G, H, Table 3) respectively, the separation between FSGS and MCD was of high significant. On the basis of the values of VIPs, p(corr), p and q the metabolites that predominantly contributed to the separation of the FSGS from the MCD group were higher levels of pyridine (PN), DMA, Pyr, and lower levels of Glc, TRG and NMN (Table 4,5).

#### 2.7 The prediction of OPLS-DA models

**Figure 4:**
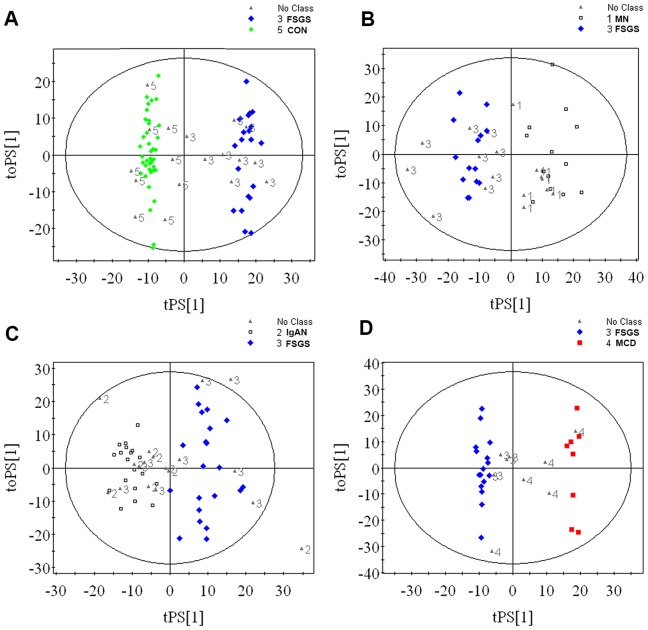
Prediction of the OPLS-DA model by non-class predictive sets. (A) FSGS-CON OPLS-DA model: sensitivity and specificity were 96.3% and 100%; (B) FSGS-MN OPLS-DA model: sensitivity and specificity were 92.6% and 96.2%; (C) FSGS-IgAN OPLS-DA model: sensitivity and specificity were 55.5% and 86.7%; (D) FSGS-MCD OPLS-DA model: sensitivity and specificity were 81.4% and 86.7%.

In the FSGS-CON OPLS-DA model, the sensitivity and specificity were 96.3% and 100% (Fig. 4A). In the FSGS-MN OPLS-DA model, the sensitivity and specificity of FSGS prediction were 92.6% and 96.2%(Fig. 4B); In FSGS-IgAN prediction model, sensitivity and specificity of FSGS prediction were 55.5% and 86.7% (Fig. 4C); In FSGS-MCD OPLS-DA models, the sensitivity and specificity of FSGS prediction were 81.4% and 86.7% (Fig. 4D).

## Discussion

### NMR-Based Metabolomics Distinguished FSGS from Healthy Controls and Other Glomerulopathies

NMR-based metabolomics can provide global, rapid, robust and reproducible metabolite profiles concerning toxicity-related pathogenesis [13], without extensive processing of samples. The emerging field of metabolomics promises immense potential for early diagnosis, therapy monitoring and f pathogenesis of diseases. In the field of nephrology, metabolomics has been efficiently and successfully applied in renal transplantation [14], acute kidney injury (AKI) [15], renal cell carcinoma [16] and diabetic nephropathy (DN) [17]. But few studies have reported the metabolic profile of glomerular diseases including FSGS.

Renal biopsy is considered the gold standard for diagnosis of FSGS. But it has poor repeatability and many limitations in monitoring progression of diseases. Clinically, biochemical parameters, such as Scr, BUN, 24 hrUprV, *et al*, can reflect the state of glomerulopathies. But from the results of the clinical biochemistry (Table 1), FSGS cannot be distinguished from other glomerulopathies only by biochemical parameters. To determine whether NMR-based metabolomics can be used to distinguish FSGS from other glomerulopathies and healthy controls, we selected Orthogonal projections to latent structure-discriminate analysis (OPLS-DA) as the chemometric approach to analyze urinary metabolic profile of FSGS and other primary glomerulopathies.

OPLS-DA is a type of supervised classification and regression method merged with class-orthogonal methodology to augment classification performance. It combines the strengths of PLS-DA and soft independent modeling of class analogy (SIMCA) classification [18]. OPLS-DA rotates the score matrix so that the class-orthogonal variation can be separated from the class-predictive one [19] and it facilitates the overall model usability. Carrola [20] *et al* analyzed urine samples from lung cancer patients and control group by OPLS-DA. Very good discrimination between cancer and control groups was achieved by multivariate modeling of urinary profiles, and by Monte Carlo Cross Validation, the classification model showed 93% sensitivity, 94% specificity and an overall classification rate of 93.5%.

In this study, the score plot of OPLS-DA showed separation trends among different groups of patients (Fig. 2). Exceptionally, there were some overlaps between FSGS and IgAN. One possible explanation was that some IgAN patients pathologically were manifested by FSGS, which indicated similarities in metabolic profile. In order to identify the special metabolic characteristics of FSGS patients, the pairwise OPLS-DA models were established among FSGS, MN, IgAN, MCD and healthy controls (Fig. 3). In respective OPLS-DA models, FSGS can be separated from other groups with high R^2^ and Q^2^, which indicated the high quality of the model. In order to confirm our results, we furthermore test the validation of every model. The FSGS-CON OPLS-DA model showed 96.3% sensitivity and 100% specificity in differentiating FSGS from healthy controls (Fig. 4A). In addition, the FSGS-MN, FSGS-MCD and FSGS-IgAN OPLS-DA models also performed good sensitivity and specificity (Fig. 4B, C,D). All the results indicated that NMR-based metabolomics analyzed by OPLS-DA is a potential non-invasive method for the differentiated diagnosis of glomerulopathies. And the external validation study confirmed the possibility of the clinical utility of the metabonomic platform in diagnosing FSGS, as well as other glomerulopathies.

### Metabolic Profile of FSGS Revealed by NMR-Based Metabolomics

Urine composition could reflect kidney function because its source of origin and metabolites might provide some clues for the pathogenesis. For the composition in urine, we found several possible metabolic pathways involving in the pathogenesis of primary FSGS.

Pyr, Cit, Ile and Val decreased, and Glc increased in patients with primary FSGS. Transporters for glucose and Krebs cycle intermediates have been identified in the brush border membrane of the tubuli [21], [22]. Krebs cycle intermediates are imported from urine by the sodium-dicarboxylate symporter NaDC-3 transporter which is mostly located in the proximal tubular cells in the kidney [23]. Additionally, glucose and Krebs cycle intermediates imported from urine could be used as energy substrates by proximal tubular cells [24]. In the present study, decreased Pyr, Cit, Ile, Val, and Tyr, important intermediates of Krebs cycle, and increased glucose indicated that the proximal tubular cells could not use glucose as an energy substrate as usual and have to compensate by importing more Krebs cycle intermediates from the urine in patients with primary FSGS.

DMA and TMA increased in FSGS patients compared with healthy controls. Increased level of DMA and TMA indicated the up-regulation of the methylamine methylamine. During the methylamine metabolism, semicarbazide-sensitive amine oxidase (SSAO) mediated deamination occurs, which directly increase oxidative stress, initiate endothelial injury and plaque formation [25]. Additionally, DMA is one of the most important metabolites of asymmetric dimethylarginine (ADMA) which is a competitive inhibitor of nitric oxide synthase (NOS) and may decrease NO availability [26]. ADMA could also contribute to oxidative stress by causing endothelial nitric oxide synthase (eNOS) uncoupling [27]. In another study, Musante, *et al*, used complementary liquid chromatography electron spray ionization tandem mass spectrometry (LC-ESI-MS/MS) and biochemical methods to analyze the serum from the patients with idiopathic FSGS, and demonstrated that albumin oxidation seems to be specific for FSGS, suggesting some pathogenetic implications [28]. In present study, increased levels of DMA and TMA indicated the existence of oxidative stress in FSGS patients.

In addition, levels of Hip decreased in patients with FSGS. Hip is formed via the conjugation of benzoate with glycine, which occurs in the kidney [29], liver [30] and intestine [31]. It is a harmful uremic toxin, and the accumulation in blood is responsible for a variety of pathological conditions. Active tubular secretion is the primary route for elimination of Hip from the plasma via the kidney and functional failure of this system causes accumulation of it in blood [32], [33]. In patients with uremia, level of Hip in serum is markedly elevated [34]. In the present study, decreased level of Hip in FSGS patients’ urine indicated the dysfunction of tubular secretion.

Finally, urinary 3-me-His and PAG levels decreased in FSGS patients, which indicated the decreased catabolism of muscle protein and lipid. 3-me-His is an important constituent bounding to the muscle proteins actin and myosin [35], and breakdown of these proteins consequently results in urinary excretion of 3-methylhistidine [36], [37]. Previous studies have reported that UPLC/MS-MS method is suitable for the determination of urinary 3-methylhistidine to monitor muscle protein catabolism [38]. PAG is an acyl glycine and it is a glycine conjugate of phenylacetic acid, which is normally a minor metabolite of fatty acids [39], so decreased level of PAG indicated the decreased catabolism of lipid.

Compared with other primary glomerular diseases (Table 4), levels of NMN decreased significantly in group of FSGS. NMN is metabolites of nicotinic acid and nicotinamide in mammals [40]. Maiza, *et al*, demonstrated that NMN could be used to monitor renal tubular excretion [41], which is secreted by proximal tubular cells through organic cation transporters (OCTs) [42]. Therefore decreased NMN indicated the dysfunction of tubular secretion compared with other glomerulopathies.

Comparison among MCD, MN and IgAN, we also observed several metabolites (Table S4) that are significant for separation. The underlying mechanisms need to be further investigated by other of metabolomics data.

## Conclusions

NMR-based metabolomics and a multivariate statistical technique can be successfully used to distinguish primary FSGS from healthy controls and other glomerulopathies with high sensitivity and specificity. It might be a potential method for non-invasive diagnosis of primary FSGS as well as other glomerulopathies. Furthermore, it can help to reveal the underling possible mechanisms of diseases. Energy metabolism disorder, oxidation stress, dysfunction of tubular secretion and decreased catabolism of muscle protein and lipid are involved in the pathogenesis of human primary FSGS. However, the application of metabolomics on the clinical work for the diagnosis and differential diagnosis of different glomerulopathies still requires further investigation.

## Supporting Information

Figure S1 **PLS-DA model for different group (CON, FSGS, IgAN, MN, MCD).**
(TIF)

Figure S2 **PCA (A) and OPLS-DA (B) scatter plots of CON (▴) and the subgroups of FSGS patients (those with the level of urine protein (C_up_) higher (♦) than 1500 mg/24 h and lower (▪) than 1500 mg/24 h).** OPLS-DA scatter plot of CON vs. a subgroup of FSGS patient with the concentration of urine protein lower than 1500****mg/24****h(C), OPLS-DA scatter plot of CON vs. a subgroup of FSGS patient with the concentration of urine protein higher than 1500****mg/24****h (D).
(TIF)

Figure S3 **PCA (A) and OPLS-DA (B) scatter plots of CON (▴) and the subgroups of FSGS patients (CKD1+CKD2(▪) and CKD3+CKD4(♦)).** OPLS-DA scatter plot of CON vs. CKD1+CKD2 of FSGS patient (C), OPLS-DA scatter plot of CON vs. CKD3+CKD4 of FSGS patient (D).
(TIF)

Table S1 **The integral value of most siginificant metabolites in healthy control (CON), FSGS, IgAN, MCD and MN patients obtained from 1H NMR spectra.**
(TIF)

Table S2 **The median and interquartile range of metabolites in each group.**
(TIF)

Table S3 **The integral change trends in subgroup analysis and the values of q, p and FDR.**
(TIF)

Table S4 **The integral change trends in MN vs. CON, IgAN vs. CON, MCD vs. CON, MN vs. IgAN, MN vs. MCD, IgAN vs. MCD, and the values of q, p and FDR.**
(TIF)
