# Supplementary figures and images for: Distinct Metabolic Profile of Primary Focal Segmental Glomerulosclerosis Revealed by NMR-Based Metabolomics
Source: PLoS One. 2013 Nov 11;8(11):e78531. doi: 10.1371/journal.pone.0078531 (PMC3823857; doi:10.1371/journal.pone.0078531)

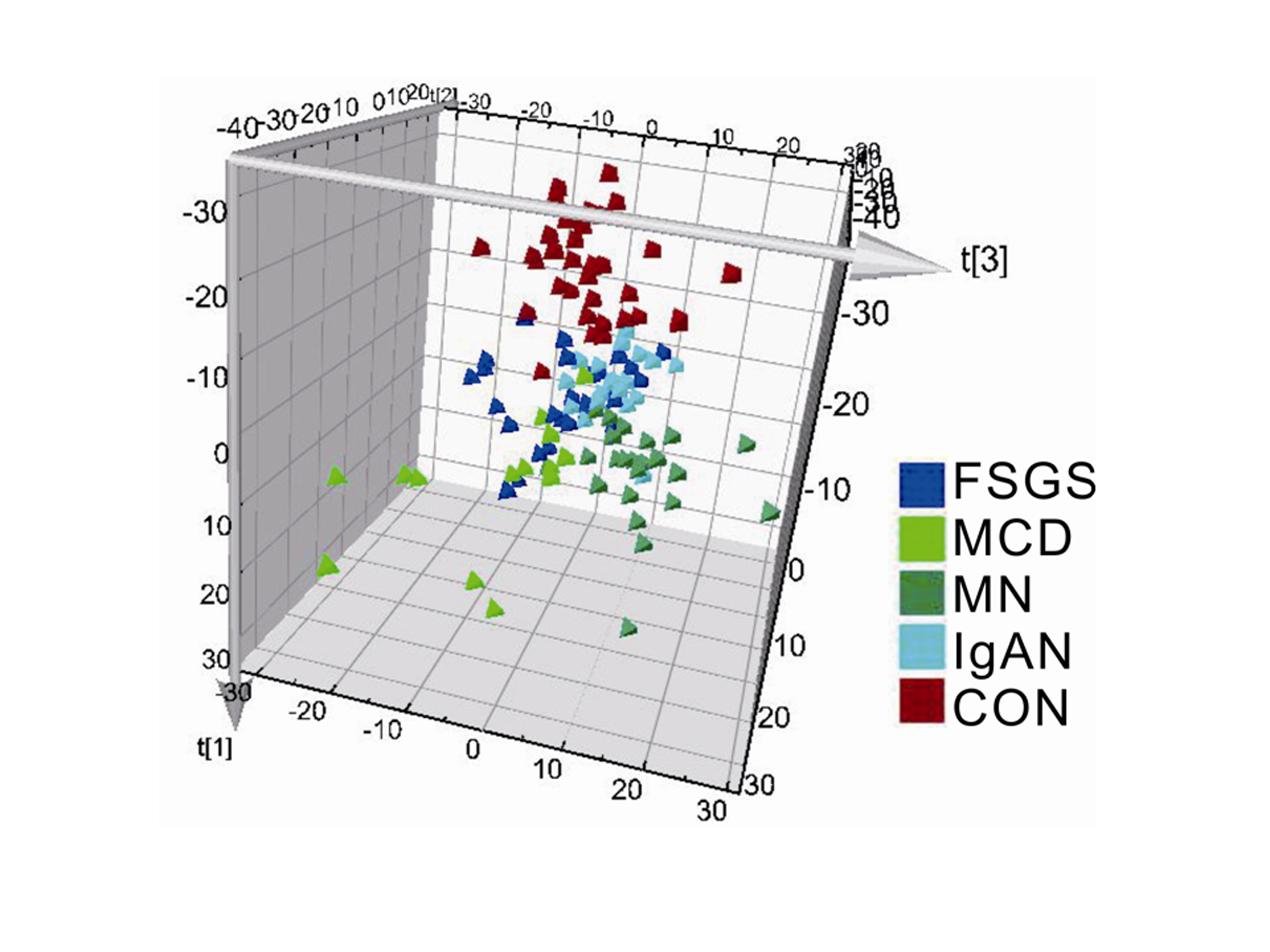

Supplement: Figure S1 — PLS-DA model for different group (CON, FSGS, IgAN, MN, MCD). (TIF) [file pone.0078531.s001.tif]

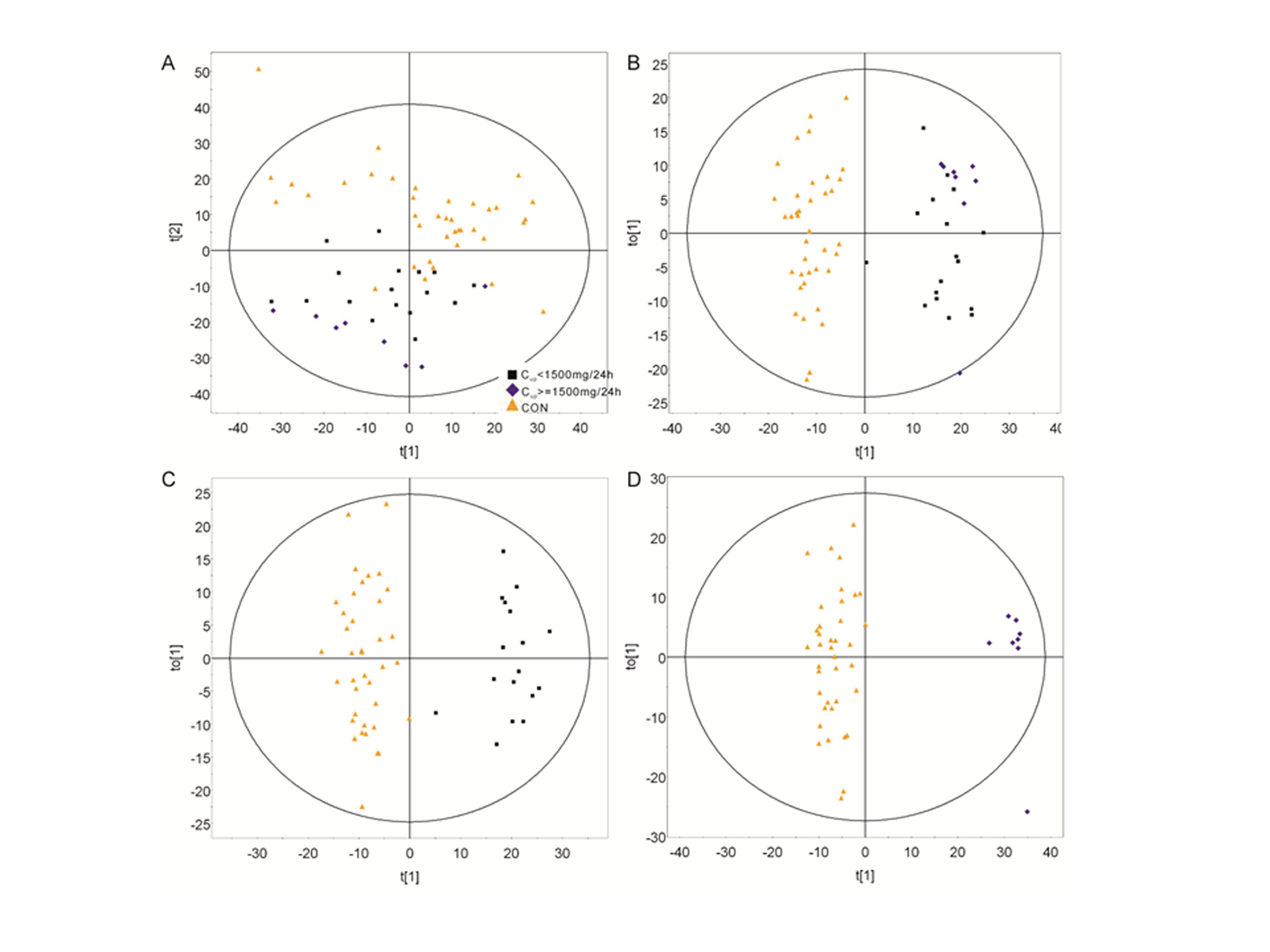

Supplement: Figure S2 — PCA (A) and OPLS-DA (B) scatter plots of CON (▴) and the subgroups of FSGS patients (those with the level of urine protein (Cup) higher (♦) than 1500 mg/24 h and lower (▪) than 1500 mg/24 h). OPLS-DA scatter plot of CON vs. a subgroup of FSGS patient with the concentration of urine protein lower than 1500mg/24h(C), OPLS-DA scatter plot of CON vs. a subgroup of FSGS patient with the concentration of urine protein higher than 1500mg/24h (D). (TIF) [file pone.0078531.s002.tif]

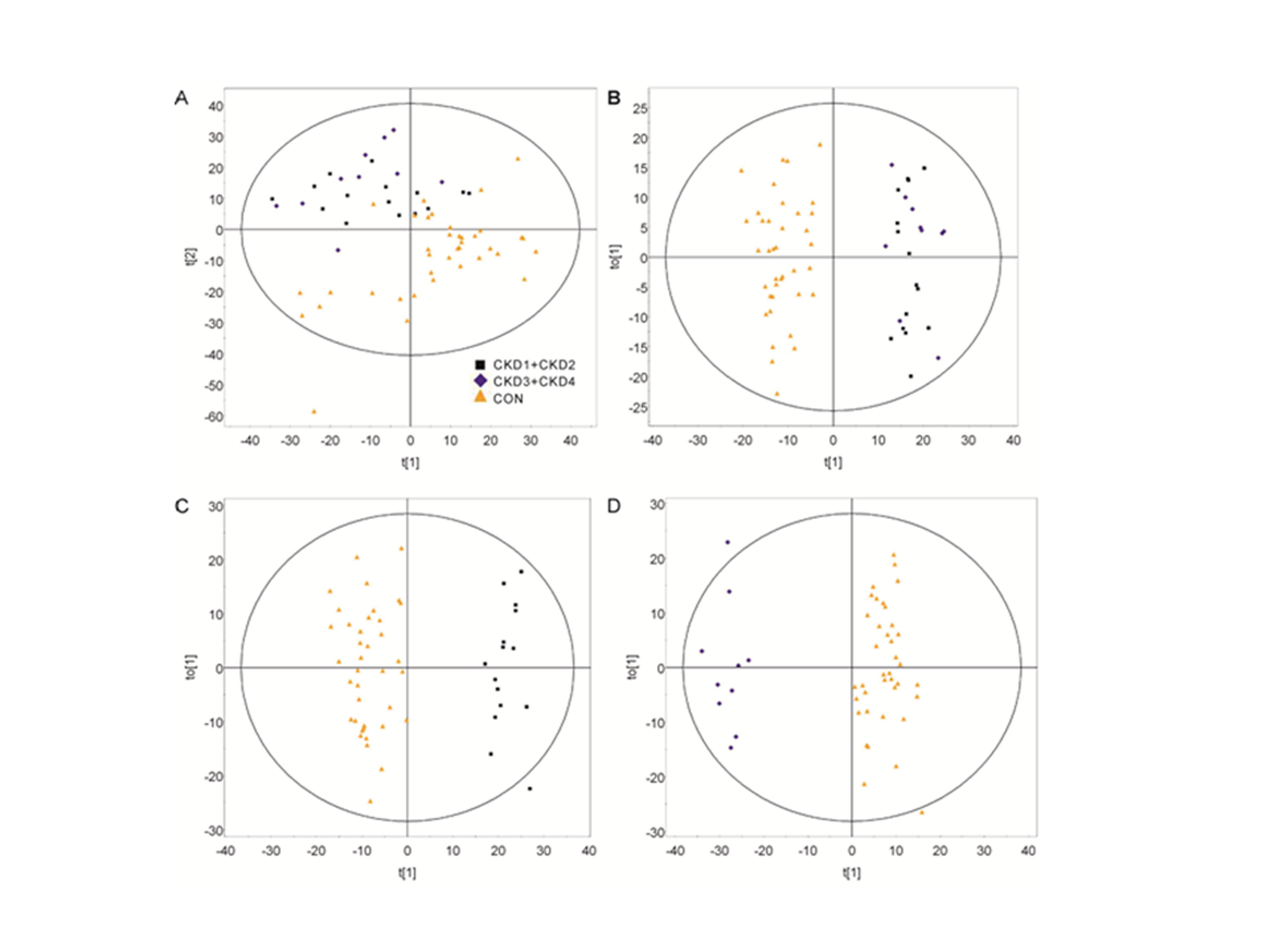

Supplement: Figure S3 — PCA (A) and OPLS-DA (B) scatter plots of CON (▴) and the subgroups of FSGS patients (CKD1+CKD2(▪) and CKD3+CKD4(♦)). OPLS-DA scatter plot of CON vs. CKD1+CKD2 of FSGS patient (C), OPLS-DA scatter plot of CON vs. CKD3+CKD4 of FSGS patient (D). (TIF) [file pone.0078531.s003.tif]

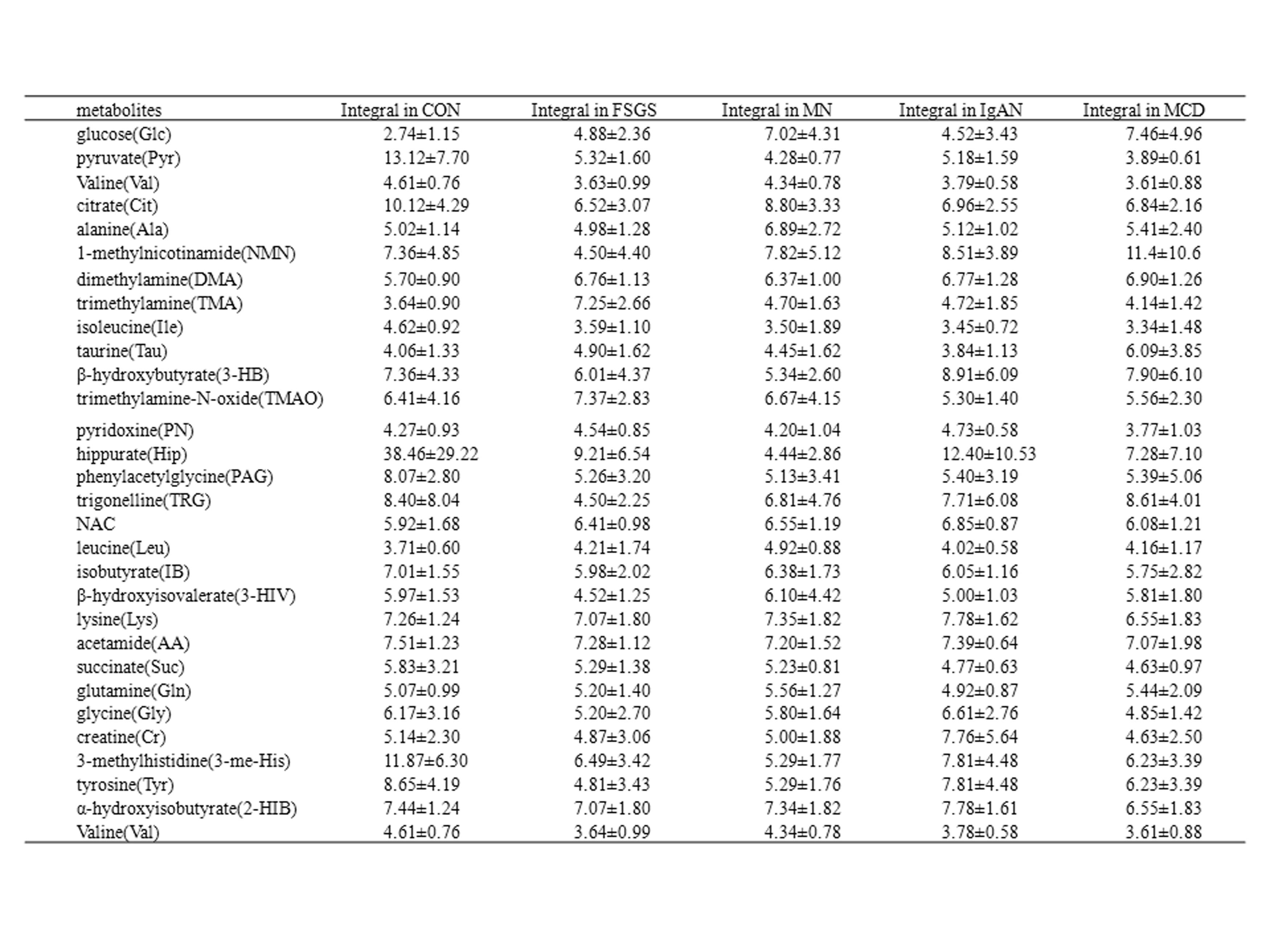

Supplement: Table S1 — The integral value of most siginificant metabolites in healthy control (CON), FSGS, IgAN, MCD and MN patients obtained from 1H NMR spectra. (TIF) [file pone.0078531.s004.tif]

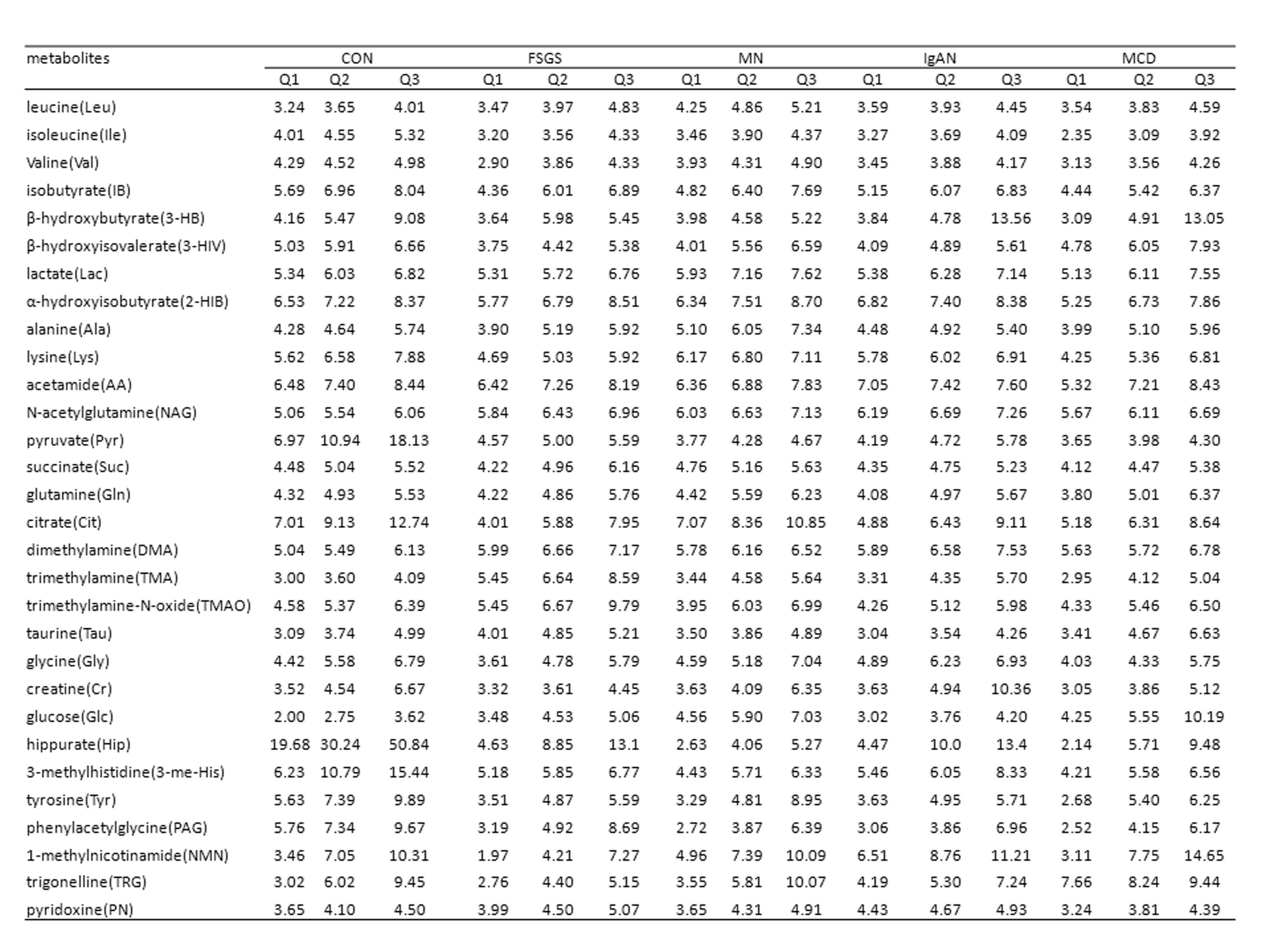

Supplement: Table S2 — The median and interquartile range of metabolites in each group. (TIF) [file pone.0078531.s005.tif]

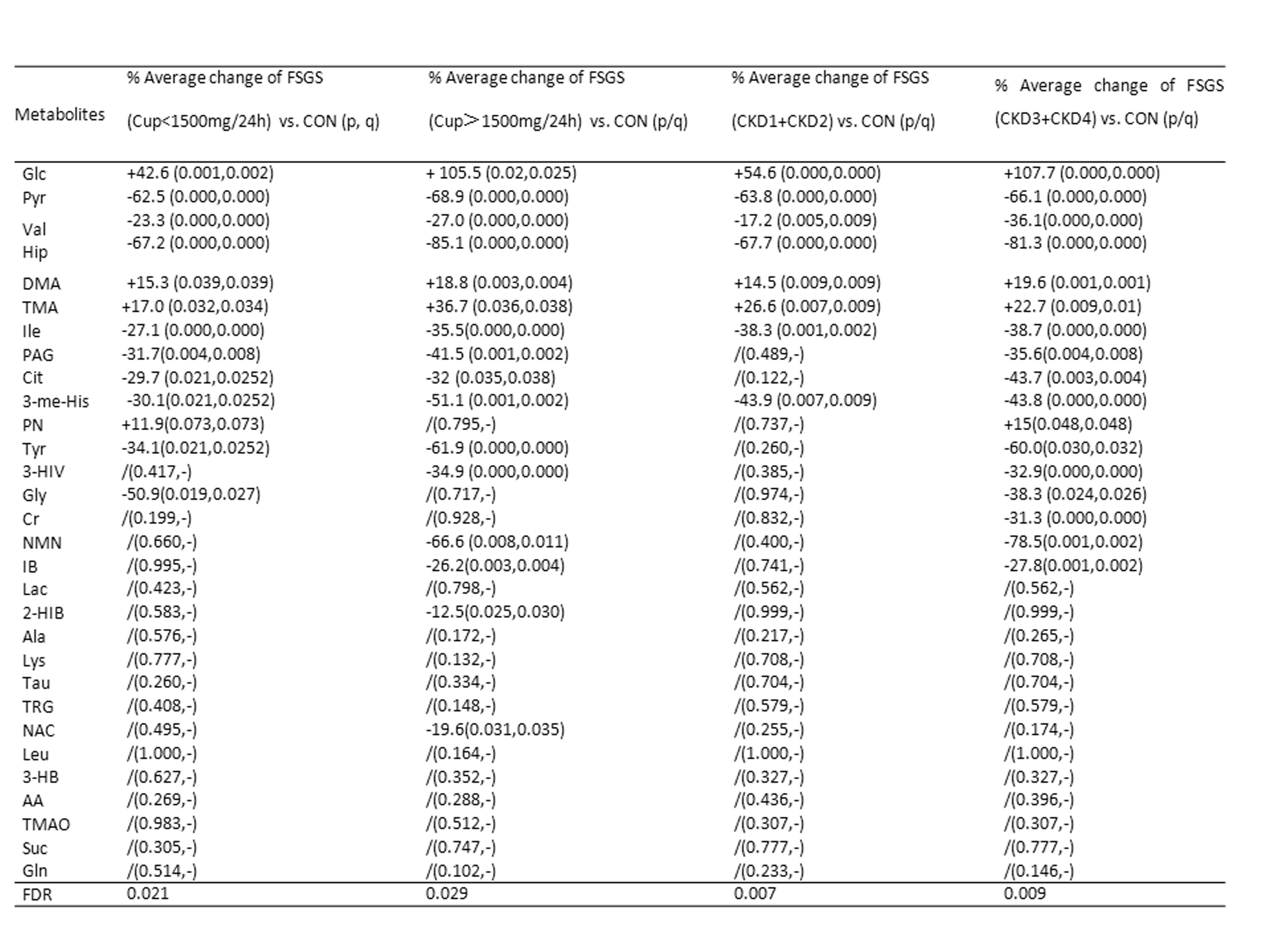

Supplement: Table S3 — The integral change trends in subgroup analysis and the values of q, p and FDR. (TIF) [file pone.0078531.s006.tif]

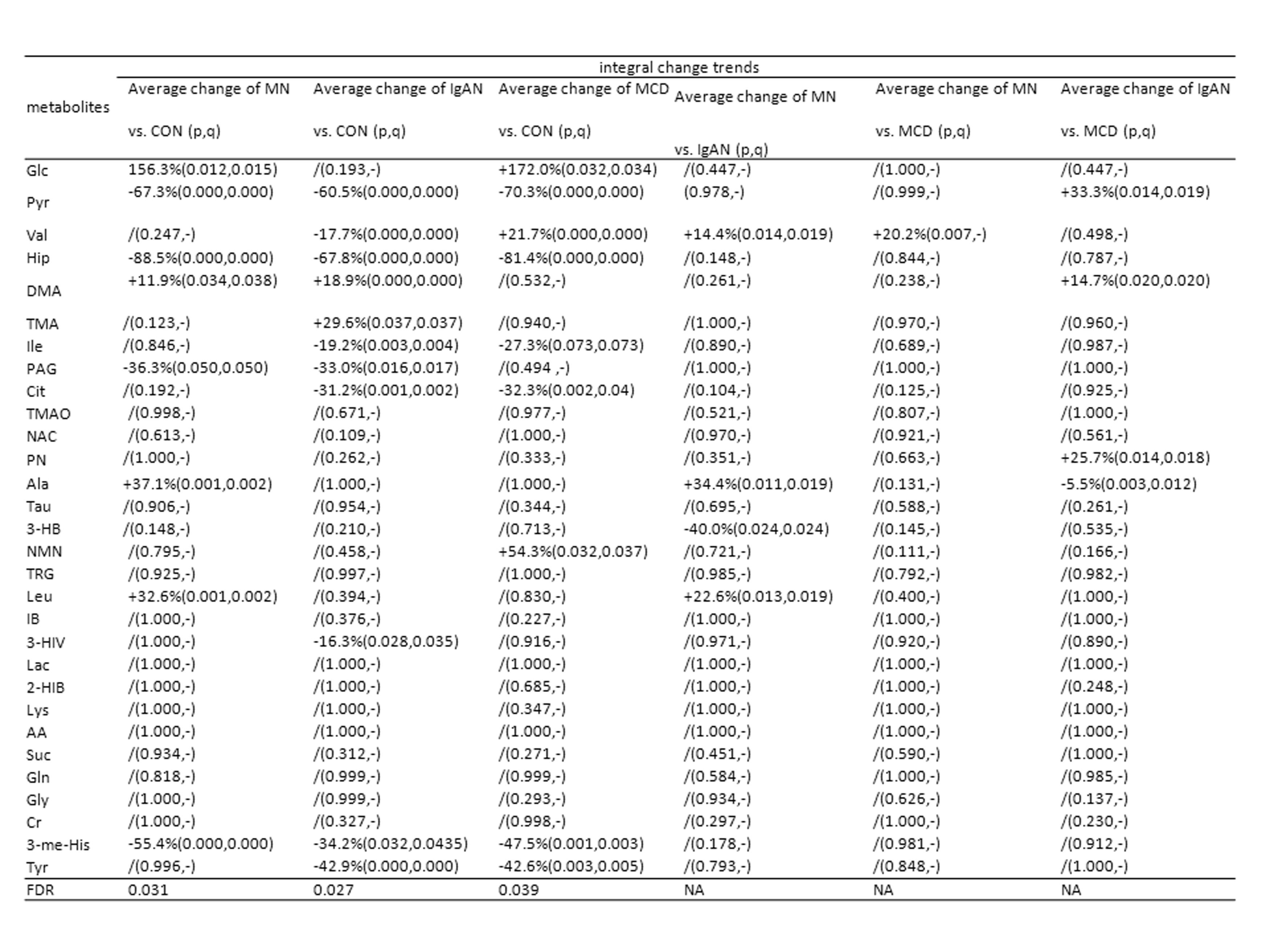

Supplement: Table S4 — The integral change trends in MN vs. CON, IgAN vs. CON, MCD vs. CON, MN vs. IgAN, MN vs. MCD, IgAN vs. MCD, and the values of q, p and FDR. (TIF) [file pone.0078531.s007.tif]
